# Tests of Educable Capacity

**Published:** 1924-10

**Authors:** 


### "TESTS OF EDUCABLE CAPACITY."

What use can be made in the public system of education of psychological tests of educable capacity ? It is an unfortunate fact, whatever the educational optimists may say, that all children are not potentially capable of the same amount of mental development. Therefore, it is with great interest that we read the report of the Consultative Committee on educable capacity, which has just been issued from the Stationery Office. Until a few years ago, the majority of investigational work in this direction was concerned with measurement of one particular ability only—intelligence. And even now for the more specific intellectual functions—those underlying memory, attention and the like, for the more important qualities of temperament, emotion and mental character and for native aptitudes—the few relevant researches have as yet yielded hardly a single simple yet trustworthy test. It seems, in fact, that in selecting the best children for higher training we still have to be satisfied with the intelligence test. But what is " intelligence " ? And what do " intelligence " tests measure ? A considerable part of the report is devoted to these questions, and they are uncommonly hard to answer. At least it is agreed that intelligence does not cover temperament and character, and that, therefore, the important personal qualities of will, feeling and emotion are not dealt with by the present tests. Also, they do not estimate acquired attainments, such as what has been learnt during reading. Nevertheless, it seems that with the present well-known school methods we must, except when the work is done by specialised experts, rest contented until more experience is gained. Apparently, no psychological tests are yet satisfactory for general use, but we can for simple school purposes employ means which estimate, in the words of the report, inborn, all-round, intellectual ability.

